# Authentic leadership and organizational citizenship behavior: the mediating role of work engagement in a Confucian cultural context

**DOI:** 10.3389/fpsyg.2026.1901087

**Published:** 2026-08-19

**Authors:** Van Dat Tran, Minh Tuan Lam Le, Thi Hao Nguyen, Pham Ngoc Thien Nguyen, Minh Vu Tran, Huynh Thuan Nguyen

**Affiliations:** 1 Faculty of Educattion, An Giang University, Vietnam National University Ho Chi Minh City, Ho Chi Minh City, Vietnam; 2 Department of Science and Technology, An Giang University, Vietnam National University Ho Chi Minh City, Ho Chi Minh City, Vietnam; 3 University of Social Sciences and Humanities, Viet Nam National University Ho Chi Minh City, Ho Chi Minh City, Vietnam; 4 Personnel Office, An Giang University, Vietnam National University Ho Chi Minh City, Ho Chi Minh City, Vietnam

**Keywords:** authentic leadership, Confucian context, organizational citizenship behavior, Vietnamese higher education, work engagement

## Abstract

**Introduction:**

In contemporary higher education, particularly within Confucian cultural contexts characterized by high power distance and hierarchical structures, leadership plays a critical role in fostering positive employee behaviors. Despite growing interest in authentic leadership, limited research has examined its influence on organizational citizenship behavior in non-Western settings, especially considering underlying mechanisms and boundary conditions. This study addresses this gap by investigating the relationship between authentic leadership and organizational citizenship behavior, with work engagement as a mediating mechanism and education level and work experience as moderating variables in Vietnamese higher education institutions.

**Methods:**

A quantitative cross-sectional design was employed, collecting data from 542 lecturers at 10 public universities in Southern Vietnam. Data were analyzed using regression-based techniques and mediation–moderation analysis.

**Results and discussion:**

The findings reveal that authentic leadership has a significant positive effect on organizational citizenship behavior and that work engagement partially mediates this relationship. However, the moderating effects of education levels and work experience were not statistically significant. These results highlight the importance of authentic leadership in enhancing engagement and promoting discretionary behaviors among academic staff, regardless of demographic differences. Practically, the study suggests that higher education institutions should prioritize leadership development initiatives that emphasize ethical behavior, transparency, and relational integrity to strengthen engagement and institutional effectiveness.

## 1 Introduction

In contemporary volatile, uncertain, complex, and ambiguous environments, leadership has become a central determinant of organizational effectiveness, particularly in knowledge-intensive sectors such as higher education (Ahmed, 2024; Jun et al., 2023; Maheshwari, 2021). Universities increasingly depend on human capital not only for knowledge creation but also for sustaining innovation, adaptability, and institutional resilience. Within this context, discretionary employee behaviors commonly conceptualized as organizational citizenship behavior play a crucial role in enhancing organizational functioning beyond formal job requirements (Organ et al., 2006; Podsakoff et al., 2003). A substantial body of research demonstrates that organizational citizenship behavior contributes to improved organizational performance, collaborative climates, and long-term sustainability (Wang and Ding, 2024; Zeinabadi, 2010; Zhang et al., 2017). Higher education increasingly recognizes engagement-driven citizenship behaviors as crucial for fostering innovation and institutional transformation, especially in transitional and emerging economies.

The Vietnamese higher education system provides a particularly relevant context for examining these dynamics. Rooted in Confucian traditions, organizational structures are typically characterized by hierarchical relationships, respect for authority, and centralized decision-making (Hallinger et al., 2017; Nguyen et al., 2015; Truong et al., 2017). Simultaneously, the sector is undergoing substantial reforms aimed at enhancing institutional autonomy, accountability, and global competitiveness (Le, 2024; Nguyen P. D. et al., 2023). The coexistence of traditional cultural norms and modern governance reforms creates a complex leadership environment that requires navigating competing expectations. Empirical evidence suggests that leadership practices in Vietnamese higher education are shaped by this tension, influencing employee engagement, relational dynamics, and citizenship behaviors (Ho and Le, 2023; Nguyen P. D. et al., 2023). However, existing research has largely focused on transformational and transactional leadership, demonstrating their positive effects on teacher satisfaction, organizational commitment, school culture, and self-efficacy (Maheshwari, 2021; Tran, 2024; Tran et al., 2023; Tran et al., 2024). Other studies highlight persistent structural challenges, including gender disparities and leadership barriers (Maheshwari, 2023; Maheshwari and Nayak, 2022), as well as links between leadership and public service motivation (Trinh et al., 2025). Despite these advances, the role of authentic leadership in this context remains underexplored.

Authentic leadership, grounded in positive organizational behavior (Luthans, 2002), emphasizes self-awareness, relational transparency, balanced processing of information, and an internalized moral perspective (Avolio and Gardner, 2005; Gardner et al., 2011; Walumbwa et al., 2008). This leadership approach is particularly relevant in high power-distance and relational cultures, where trust, ethical consistency, and interpersonal authenticity are essential for effective leader–follower relationships. Empirical studies consistently show that authentic leadership enhances employee engagement, wellbeing, and positive work attitudes (Alok and Israel, 2012; Rego et al., 2012; Wirawan et al., 2020; Zhang et al., 2022). In educational settings, it has been linked to increased teacher motivation, organizational commitment, and professional wellbeing (Xu and Pang, 2024). Furthermore, emerging evidence suggests that authentic leadership promotes organizational citizenship behavior indirectly through affective and relational mechanisms such as trust, identification, and engagement (Farid et al., 2020; Hirst et al., 2016). Studies in Confucian-influenced contexts further indicate that leadership styles emphasizing moral integrity and relational harmony can strengthen both engagement and citizenship behaviors (Nguyen et al., 2024; Yen et al., 2024).

Despite the increasing evidence supporting the positive link between authentic leadership and organizational citizenship behavior, the underlying motivational mechanisms are not sufficiently integrated into a cohesive theoretical framework. Social exchange theory suggests that employees reciprocate supportive and ethical leadership with discretionary contributions (Blau, 1964). Authentic leaders cultivate high-quality exchange relationships marked by trust and integrity, which encourage organizational citizenship behavior (Avolio et al., 2004). Additionally, self-determination theory elucidates how leadership affects intrinsic motivation by fulfilling employees’ needs for autonomy, competence, and relatedness (Deci and Ryan, 2008; Ryan and Deci, 2001, 2011). Authentic leadership behaviors particularly transparency and ethical consistency can foster autonomous motivation thereby enhancing engagement and promoting citizenship behaviors (Meyer and Gagné, 2008). However, empirical models that combine social exchange theory and self-determination theory to elucidate the relationship between authentic leadership, engagement, and organizational citizenship behavior are still limited, particularly within higher education contexts.

Leadership effects are rarely uniform across individuals, highlighting the importance of examining boundary conditions. Education level and work experience are particularly relevant individual characteristics that shape employees’ cognitive schemas, expectations, and interpretations of leadership behaviors (Ariffin and Norbani Che, 2014; Ayouz et al., 2025; Blau, 1964; Hadi et al., 2023; Hitka et al., 2021; Hoefsmit et al., 2023; Liang et al., 2014; Liu et al., 2018). Highly educated employees may evaluate leadership authenticity more critically, while more experienced employees may rely on accumulated relational and contextual knowledge when interpreting leader behaviors. Additionally, generational and career-stage differences influence motivational responses to leadership styles (Zacher and Schmitt, 2016). Although prior research has identified moderators of authentic leadership outcomes (Zhang et al., 2022), the specific moderating roles of education level and work experience within the authentic leadership–engagement–organizational citizenship behavior pathway remain underexplored.

The need for such investigation is particularly salient in non-Western contexts. The predominance of authentic leadership research in Western settings raises questions about its cross-cultural applicability (Polat et al., 2024). In Confucian-influenced societies such as Vietnam, hierarchical structures, collectivist norms, and moral expectations shape leadership practices and employee behaviors (Hallinger et al., 2017; Nguyen et al., 2015; Truong et al., 2017). At the same time, higher education reforms emphasizing autonomy and accountability introduce new expectations for transparency, ethical governance, and employee engagement (Le, 2024; Nguyen P. D. et al., 2023). Emerging evidence indicates that Confucian values significantly influence organizational commitment, moral identity, and citizenship behaviors in Vietnamese institutions (Nguyen et al., 2024). These dynamics suggest that authentic leadership may be particularly relevant, yet its effectiveness may depend on both cultural and individual factors.

Although alternative leadership styles, such as benevolent and servant leadership, have been examined in Vietnam, demonstrating positive effects on organizational citizenship behavior through relational and psychological mechanisms (Ho and Le, 2023; Nguyen P. D. et al., 2023; Nguyen et al., 2025; Tran Q. H. N, 2023; Tran, 2025a), authentic leadership remains relatively underexamined. Existing studies from related contexts suggest that authentic leadership enhances organizational citizenship behavior through affective and motivational pathways, including forms of work passion and engagement (Yen et al., 2024). However, its role within Vietnamese higher education institutions has not been sufficiently theorized or empirically validated. Addressing this gap is critical given increasing demands for ethical leadership and sustainable organizational performance. Accordingly, this study investigates the relationship between authentic leadership and organizational citizenship behavior, with work engagement as a mediating mechanism, in Vietnamese higher education institutions. It further examines the moderating roles of education levels and work experience in shaping these relationships. By integrating theories within a unified framework and incorporating cultural and individual contingency perspectives, this study contributes to the cross-cultural validation of authentic leadership and extends understanding of its applicability in high power-distance, Confucian contexts. Practically, it offers insights into how leadership practices can be tailored to diverse academic staff profiles to enhance engagement and promote citizenship behaviors essential for institutional effectiveness. Specifically, the study addresses the following research questions:

*RQ1*: To what extent is authentic leadership associated with organizational citizenship behavior among university lecturers?

*RQ2*: Does work engagement mediate the relationship between authentic leadership and organizational citizenship behavior?

*RQ3*: Does education level moderate the relationship between authentic leadership and work engagement?

*RQ4*: Does work experience moderate the relationship between authentic leadership and organizational citizenship behavior?

## 2 Literature review and hypotheses development

### 2.1 Theoretical framework

**FIGURE 1:**
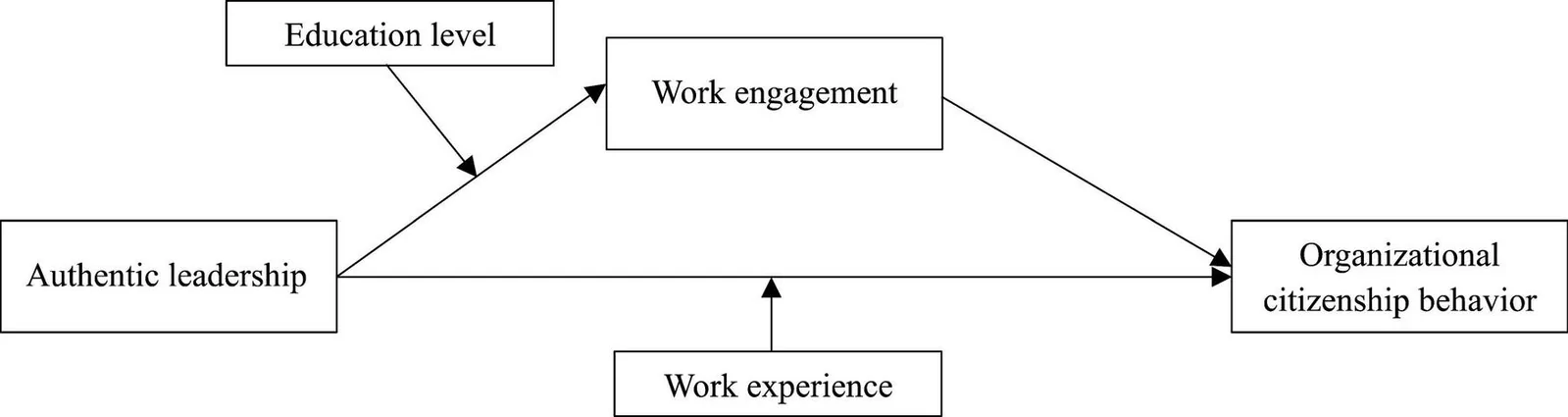
The theoretical framework derived from theories.

The present study examines whether the relationships among authentic leadership, work engagement, and organizational citizenship behavior differ according to education level and work experience (Figure 1). To address this question, the study integrates complementary theoretical perspectives, including authentic leadership theory (Walumbwa et al., 2008), broaden-and-build theory (Fredrickson, 2001), the job demands–resources model (Bakker and Demerouti, 2007, 2017), social exchange theory (Blau, 1964), and social learning theory (Bandura, 1977). These frameworks explain the motivational mechanisms and contextual contingencies through which leadership shapes employee behavior in higher education.

Authentic leadership theory conceptualizes leadership as a pattern of behavior characterized by self-awareness, relational transparency, internalized moral perspective, and balanced processing (Walumbwa et al., 2008). These attributes foster trust, ethical consistency, and high-quality relationships, which are particularly salient in collectivist and high power-distance contexts. Empirical evidence indicates that such leadership promotes discretionary behaviors, including organizational citizenship behavior (Yen et al., 2024), with similar findings reported in Vietnamese higher education settings (Ho and Le, 2023; Nguyen P. D. et al., 2023; Nguyen et al., 2025; Tran Q. H. N, 2023; Tran, 2025b). Social exchange theory provides the primary mechanism linking authentic leadership to organizational citizenship behavior. Employees reciprocate through extra-role behaviors when they perceive their leaders as fair and genuine (Blau, 1964). Strong leader-member relationships reinforce this process (Ho and Le, 2023; Nguyen P. D. et al., 2023). Complementing these findings, social learning theory explains how leaders act as role models whose ethical conduct is observed and internalized, shaping employees’ behavioral norms (Bandura, 1977). The job demands–resources model further explains the mediating role of work engagement (Bakker and Demerouti, 2007, 2017). Authentic leadership functions as a key job resource that enhances employees’ vigor, dedication, and absorption, thereby motivating discretionary effort and organizational citizenship behavior. Empirical studies in Vietnamese contexts support the link between leadership, engagement, and citizenship outcomes (Nguyen et al., 2025; Tran Q. H. N, 2023; Tran, 2025b), while relational and ethical leadership styles have been shown to strengthen engagement (Nguyen et al., 2024; Yen et al., 2024). Importantly, education level and work experience are introduced as moderating variables. Drawing on broaden-and-build theory, these characteristics represent personal resources that shape how individuals interpret leadership and translate engagement into behavior (Fredrickson, 2001). Education improves cognitive evaluation, while work experience improves relational understanding. This means that leadership effectiveness may vary between different groups. Taken together, these perspectives form a moderated-mediation framework in which authentic leadership predicts organizational citizenship behavior through work engagement. Education level moderates the leadership–engagement relationship, while work experience moderates the leadership–organizational citizenship behavior link. This integrated approach offers a nuanced explanation of how, why, and for whom authentic leadership fosters organizational citizenship behavior in higher education.

### 2.2 Authentic leadership and organizational citizenship behavior

Authentic leadership has become a central construct in organizational behavior, particularly in response to ethical failures and declining trust in leadership (Gardner et al., 2011). Rooted in positive psychology, it refers to a pattern of leader behavior that promotes positive psychological capacities and an ethical organizational climate (Luthans and Avolio, 2003; Walumbwa et al., 2008). The construct comprises four dimensions: self-awareness, balanced processing, internalized moral perspective, and relational transparency (Avolio and Gardner, 2005; Walumbwa et al., 2008). These dimensions reflect leaders’ capacity for ethical self-regulation, objective decision-making, and openness in relationships. Unlike authority-driven approaches, authentic leadership relies on integrity and consistency to influence followers (Avolio et al., 2004), fostering trust, ethical conduct, and strong leader–follower relationships (Northouse, 2021; Wu and Chen, 2019). Such qualities are particularly important in higher education, where collaboration and values-driven performance are essential.

Organizational citizenship behavior, in contrast, refers to discretionary actions that go beyond formal job requirements and contribute to organizational effectiveness (Organ et al., 2006). Originating from Katz’s (1964) concept of extra-role behavior, organizational citizenship behavior includes voluntary activities that are not formally rewarded but are vital for organizational functioning (Organ, 1977; Smith et al., 1983). It encompasses dimensions such as altruism, conscientiousness, sportsmanship, courtesy, and civic virtue, as well as expanded forms including helping behavior, loyalty, and initiative (Podsakoff et al., 2003). A common distinction differentiates organizational citizenship behavior directed toward individuals from that directed toward the organization (Eliyahu and Somech, 2023; Lee and Allen, 2002). These behaviors enhance cooperation, productivity, and long-term sustainability (Lin and Ho, 2010).

Extensive research demonstrates a positive relationship between leadership and organizational citizenship behavior, with meta-analytic evidence indicating effect sizes ranging from *r* = 0.09 to *r* = 0.35 (Podsakoff et al., 2003). Authentic leadership is particularly influential due to its emphasis on ethical integrity and relational transparency (Avolio and Gardner, 2005; Avolio et al., 2004; Luthans and Avolio, 2003). These attributes enhance trust, satisfaction, and commitment, encouraging employees to engage in discretionary behaviors. Theoretical explanations highlight mechanisms such as role modeling, emotional contagion, and social identification (Ilies et al., 2005). Empirical studies consistently support this relationship across contexts, including education and non-Western settings (Alvarez et al., 2019; Asif et al., 2025; Iqbal et al., 2018; Jun et al., 2023; Shapira-Lishchinsky and Tsemach, 2014). For example, Asif et al. (2025) show that authentic leadership mitigates the negative effects of organizational politics while enhancing organizational citizenship behavior, whereas Iqbal et al. (2018) highlight the strengthening role of procedural justice. In educational contexts, Walumbwa et al. (2010) report a significant positive effect (β = 0.20, *p* < 0.01), with identification and empowerment as key mechanisms. Additional studies identify mediating roles of trust, commitment, and identification (Farid et al., 2020; Joo and Jo, 2017; Shie and Chang, 2022). Beyond direct effects, authentic leadership influences organizational citizenship behavior through psychological mechanisms such as work engagement, trust, empowerment, and identification (Jun et al., 2023; Qiu et al., 2019; Shapira-Lishchinsky and Tsemach, 2014; Wei et al., 2018). Drawing on social exchange theory and social learning theory, employees reciprocate ethical leadership and internalize observed behaviors, reinforcing organizational citizenship behavior (Bandura, 1977; Blau, 1964). Building on this foundation, the present study proposes:

*H1*: Authentic leadership is positively associated with the organizational citizenship behavior of faculty members.

### 2.3 Mediating role of work engagement

Work engagement is widely conceptualized as a positive, fulfilling, and work-related psychological state characterized by vigor, dedication, and absorption (Bakker et al., 2011; Burić and Macuka, 2018; Li et al., 2022; Meyer and Gagné, 2008; Salanova et al., 2005; Schaufeli, 2013; Schaufeli and Bakker, 2010). It reflects a condition in which individuals experience high levels of energy, strong involvement, and deep immersion in their work (Schaufeli, 2013; Schaufeli and Bakker, 2010). The Utrecht Work Engagement Scale operationalizes this construct across three dimensions: vigor, referring to energy and persistence; dedication, reflecting enthusiasm and a sense of significance; and absorption, indicating full concentration and immersion in tasks (Schaufeli et al., 2006; Schaufeli, 2013). Together, these dimensions highlight engagement as a multidimensional construct that enhances both employee wellbeing and organizational effectiveness. In educational settings, teacher engagement is strongly associated with instructional quality, professional commitment, and student outcomes (Burić and Macuka, 2018; Li et al., 2022; Minghui et al., 2018; Wang, 2022).

Empirical evidence further indicates that work engagement is positively linked to self-efficacy, organizational commitment, and access to job resources, all of which contribute to improved performance and wellbeing (Baquero, 2023; Burić and Macuka, 2018; Chen et al., 2024; Fitriasari and Ummah, 2020; Li et al., 2022; Liu and Huang, 2019; Maksum et al., 2022; Minghui et al., 2018; Miraflor and Lyndon, 2022; Moreira-Fontán et al., 2019; Tran V. D., 2023; Wang, 2022). From a theoretical perspective, self-determination theory emphasizes the role of intrinsic motivation in fostering engagement (Meyer and Gagné, 2008), while broader organizational research links engagement to enhanced performance and service outcomes (Salanova et al., 2005). Importantly, a growing body of literature supports the mediating role of work engagement in the relationship between authentic leadership and positive employee outcomes. For instance, work engagement has been shown to mediate the effects of authentic leadership on innovation and creativity (Chaudhary and Panda, 2018; Lv et al., 2022), as well as on task performance and organizational citizenship behavior (Wei et al., 2018).

Within the job demands–resources model, authentic leadership can be conceptualized as a critical job resource that enhances employees’ motivational states (Bakker and Demerouti, 2007). Leadership behaviors such as transparency, ethical consistency, and individualized consideration provide psychological support that strengthens employees’ energy, dedication, and involvement. These resources foster higher levels of engagement, which in turn enable employees to invest discretionary effort beyond formal job requirements. As a result, engaged employees are more likely to exhibit organizational citizenship behavior. This perspective explains not only that authentic leadership predicts organizational citizenship behavior, but also why this relationship occurs through the development of motivational resources that sustain proactive and citizenship-oriented behaviors. Consequently, work engagement functions as a principal psychological mechanism connecting authentic leadership to organizational citizenship behavior. By fostering intrinsic motivation, psychological connection, and sustained involvement in work, authentic leadership enhances engagement, which subsequently translates into higher levels of discretionary behavior. Therefore, the following hypothesis is proposed:

*H2*: Work engagement mediates the positive association between authentic leadership and organizational citizenship behavior.

### 2.4 Moderating roles of education level and work experience

While work engagement explains how authentic leadership translates into organizational citizenship behavior, these relationships are unlikely to be uniform across employees. Individual differences particularly education level and work experience shape how employees perceive, interpret, and respond to leadership behaviors. Examining these moderators provides a more complex view of the boundary conditions under which authentic leadership is most effective.

### 2.5 Moderating role of education level

Education level is a critical individual characteristic that influences cognitive capacity, value orientation, and responsiveness to leadership. Employees with higher levels of education typically possess broader cognitive frameworks, stronger analytical abilities, and greater autonomy in problem-solving (Hadi et al., 2023; Hitka et al., 2021; Hoefsmit et al., 2023; Liang et al., 2014). These attributes are often associated with heightened sensitivity to workplace conditions, including leadership behaviors (Liang et al., 2023). Empirical evidence suggests that highly educated employees respond more strongly to leadership styles; for example, transformational leadership demonstrates stronger positive effects on job satisfaction among this group (Liu et al., 2024). Additionally, higher education is linked to stronger organizational commitment and value alignment (Ariffin and Norbani Che, 2014), indicating that such employees are more likely to engage when leadership behaviors align with expectations of autonomy, transparency, and ethical conduct.

Within this context, authentic leadership characterized by relational transparency, balanced processing, and an internalized moral perspective closely aligns with the cognitive and value orientations of highly educated employees. This alignment increases the likelihood that such leadership will be perceived as a meaningful resource, thereby strengthening work engagement and, indirectly, organizational citizenship behavior. In contrast, employees with lower levels of education may be less responsive to autonomy-supportive and empowerment-oriented leadership, instead preferring more directive approaches that provide clear structure and guidance. Supporting this view, prior research indicates that relationship-oriented leadership styles tend to have weaker effects among employees with lower education levels (Ayouz et al., 2025). Education level is expected to moderate the relationship between authentic leadership and work engagement rather than the engagement–organizational citizenship behavior link. This is because education primarily influences how leadership behaviors are cognitively appraised rather than how engagement translates into action. Highly educated employees are more likely to recognize and value leaders’ authenticity and ethical consistency, resulting in stronger engagement responses. Accordingly, the following hypothesis is proposed:

*H3*: Education level moderates the association between authentic leadership and work engagement, such that the association is stronger for employees with higher levels of education.

### 2.6 Moderating role of work experience

The moderating role of work experience in shaping the effectiveness of authentic leadership can be explained through social learning theory and social exchange theory (Bandura, 1977; Blau, 1964). From a social learning perspective, employees develop more sophisticated cognitive schemas for interpreting leadership behaviors through repeated organizational interactions. Over time, accumulated experience enhances their ability to recognize nuanced leadership signals. In parallel, social exchange theory suggests that longer tenure strengthens relational bonds, trust, and psychological contracts, thereby intensifying reciprocity between leaders and followers. Together, these perspectives indicate that more experienced employees are better equipped—both cognitively and relationally to respond to authentic leadership.

Empirical research supports this proposition. Authentic leadership has been shown to foster proactive behavior and reduce workplace deviance through mechanisms such as supervisor identification, psychological safety, and job engagement (Liu et al., 2018). Similarly, Hirst et al. (2016) demonstrate that authentic leadership influences leader–member exchange and helping behaviors through cascading relational processes. These findings suggest that the effectiveness of authentic leadership depends on employees’ capacity to recognize and internalize its ethical and relational qualities capacities that are strengthened through experience. More experienced employees typically possess a more profound understanding of organizational dynamics and leadership processes, enabling them to better interpret key features of authentic leadership, such as transparency and value-driven behavior. As a result, they are more likely to align with leaders’ values, experience stronger engagement, and translate this engagement into organizational citizenship behavior. In contrast, less experienced employees may rely more on formal structures and explicit guidance, making them less responsive to the implicit and relational nature of authentic leadership. Broaden-and-build theory further explains this effect by suggesting that accumulated emotional and relational resources expand individuals’ cognitive and behavioral repertoires (Fredrickson, 2001). Consequently, work experience enhances both the interpretation of leadership and the enactment of discretionary behaviors. Accordingly, the following hypothesis is proposed:

*H4*: Work experience moderates the association between authentic leadership and organizational citizenship behavior, such that the association is stronger for employees with higher levels of work experience.

## 3 Materials and methods

### 3.1 Research design

This study employed a quantitative cross-sectional design to examine the relationships among authentic leadership, work engagement, and organizational citizenship behavior, including the mediating role of work engagement and the moderating effects of education levels and work experience. The research was conducted in public universities in Southern Vietnam, where understanding leadership mechanisms is critical for enhancing institutional effectiveness. Cross-sectional quantitative designs are widely used in Vietnamese and comparable contexts to investigate leadership–engagement–organizational citizenship behavior relationships, particularly when testing mediation and moderation models (Cao and Dong, 2024; Nguyen P. D. et al., 2023; Tran, 2025b). While such designs provide useful insights into organizational dynamics, they are also associated with limitations in establishing causal relationships. Nonetheless, they remain appropriate for examining theoretically grounded models within specific institutional settings such as higher education.

### 3.2 Participants and sampling procedure

The study targeted lecturers from 10 public universities in the Mekong Delta region of Vietnam, with an intended sample size exceeding 610 participants. A stratified random sampling technique was employed to enhance representativeness and reduce sampling bias. Stratification was based on key demographic and professional characteristics, including gender, age, educational attainment, and years of teaching experience, in order to capture the diversity of the academic workforce. At the institutional level, universities were further stratified according to size (large, medium, and small), number of faculty members, and dominant academic disciplines (e.g., social sciences, engineering, agriculture, and medicine). Within each stratum, lecturers were selected using proportional random sampling to ensure that the sample reflected the relative size and structure of each institution. A total of 610 lecturers were invited to participate in the survey, of whom 542 returned valid responses, yielding a response rate of 88.9%. The sample consisted of 253 male lecturers (46.7%) and 289 female lecturers (53.3%). In terms of age distribution, the majority of participants were between 40 and 49 years old (44.8%), followed by those aged 30–39 years (20.8%) and those aged 50 years or above (17.2%), while lecturers aged 29 years or younger accounted for 17.2%. Regarding educational attainment, more than half of the respondents held doctoral degrees (53.5%), followed by master’s degrees (31.2%) and bachelor’s degrees (15.3%). With respect to teaching experience, the largest proportion of participants had between 16 and 30 years of experience (39.1%), followed by 6–15 years (36.5%), 5 years or less (18.1%), and 31 years or more (6.3%). Overall, these characteristics broadly reflect the demographic and professional profile of university lecturers in the Mekong Delta region. Detailed information is presented in Table 1.

**TABLE 1:** Demographic characteristics of the sample.

Variable	Category	*n*	%
Educational level	Bachelor’s degree	83	15.3
	Master’s degree	169	31.2
	Doctorate	290	53.5
Work experience	≤5 years	98	18.1
	6–15 years	198	36.5
	16–30 years	212	39.1
	≥31 years	34	6.3

*N* = 542.

### 3.3 Instruments and measures

The study examines three primary constructs authentic leadership, work engagement, and organizational citizenship behavior as presented in Figure 1. To ensure linguistic and conceptual equivalence, all instruments were translated into Vietnamese using a back-translation procedure (Brislin, 1970). Two bilingual experts independently translated the original English scales, followed by expert review for clarity, cultural relevance, and content validity. An independent translator then conducted back-translation, and discrepancies were resolved through consensus. A pilot study was conducted to assess item clarity and contextual appropriateness. Table 2 reports the confirmatory factor analysis (CFA) results and reliability coefficients for all constructs. To further examine the distinctiveness of the three constructs, a combined CFA was conducted incorporating all items from authentic leadership, work engagement, and organizational citizenship behavior simultaneously. The three-factor model demonstrated acceptable fit (χ^2^/df = 2.26, RMSEA = 0.05, CFI = 0.911, TLI = 0.904), which, together with the strong fit indices for each individual scale and satisfactory internal consistency (Cronbach’s α = 0.82–0.87), supports the empirical distinctiveness of the three constructs. Moreover, common method bias was assessed using Harman’s single-factor test. The first unrotated factor accounted for 25.39% of the total variance, which is well below the recommended threshold of 50%, suggesting that common method bias is unlikely to pose a serious threat to the study findings.

**TABLE 2:** CFA results and reliability of the study constructs.

Scale	No. of items	χ^2/^df	RMSEA	CFI	TLI	Conbach’s alpha
ALQ	16	2.23	0.04	0.961	.952	0.82
OCB	13	2.81	0.05	0.963	0.956	0.87
UWE	9	1.58	0.03	0.990	0.981	0.82
Combined three-factor measurement model	38	2.26	0.05	0.911	0.904	–

ALQ, Authentic leadership questionnaire; OCB, organizational citizenship behavior; UWE, Utrecht Work Engagement. The combined three-factor measurement model included ALQ, OCB, and WE as separate latent constructs.

#### 3.3.1 Authentic leadership

Authentic leadership was measured using the authentic leadership questionnaire developed by Walumbwa et al. (2008). The scale consists of 16 items rated on a five-point Likert scale ranging from 1 (“strongly disagree”) to 5 (“strongly agree”). The ALQ captures four dimensions: self-awareness (4 items; e.g., “Seeks feedback to improve interactions with others”), relational transparency (5 items; e.g., “Tells you the hard truth”), balanced processing (3 items; e.g., “Analyzes relevant data before coming to a decision”), and internalized moral perspective (4 items; e.g., “Makes decisions based on his or her core values”). Exploratory factor analysis (EFA) using principal axis factoring (PAF) with promax rotation supported a clear four-factor structure (KMO = 0.82; Bartlett’s χ^2^(105) = 3112.00, *p* < 0.000), explaining 55.70% of the total variance. Factor loadings were strong across dimensions: internalized moral perspective (0.73–0.80), relational transparency (0.70–0.78), self-awareness (0.67–0.77), and balanced processing (0.63–0.80). Item 9 was removed due to low loadings ( < 0.50). CFA with the remaining 15 items confirmed the four-factor structure of the Vietnamese ALQ, showing good fit indices (χ^2^ = 187.81, df = 84, χ^2^/df = 2.236, *p* = 0.000, TLI = 0.95, CFI = 0.96, RMSEA = 0.04). Standardized loadings ranged from 0.69 to 0.79. Reliability was satisfactory, with Cronbach’s alpha of 0.82 overall and subscale alphas of 0.85 (internalized moral perspective), 0.84 (relational transparency), 0.80 (self-awareness), and 0.77 (balanced processing), confirming good model fit and internal consistency.

#### 3.3.2 Work engagement

Work engagement was measured using the Utrecht Work Engagement Scale developed by Schaufeli et al. (2006). This widely used instrument comprises 9 items representing three dimensions: vigor (3 items; e.g., “At my job, I feel strong and vigorous”), dedication (3 items; e.g., “My job inspires me”), and absorption (3 items; e.g., “I am immersed in my work”). Responses were recorded on a five-point frequency scale ranging from 1 (“never”) to 5 (“very often”), adapted for consistency with the overall survey design. EFA using principal axis factoring (PAF) with promax rotation supported the 9-item scale [KMO = 0.84; Bartlett’s χ^2^(36) = 1379.07, *p* < 0.000], with three factors explaining 49.33% of the variance. All items loaded appropriately: vigor (0.51–0.81), dedication (0.60–0.74), and absorption (0.57–0.77). CFA indicated good model fit (χ^2^ = 37.99, df = 24, χ^2^/df = 1.583, *p* = 0.03, RMSEA = 0.03, CFI = 0.99, TLI = 0.98). Reliability was satisfactory (α = 0.82 overall; 0.76 vigor; 0.71 dedication; 0.72 absorption), confirming acceptable construct validity.

#### 3.3.3 Organizational citizenship behavior

Organizational citizenship behavior was measured using the 16-item scale developed by Lee and Allen (2002), rated on a five-point Likert scale ranging from 1 (“strongly disagree”) to 5 (“strongly agree”). The scale includes two dimensions: organizational citizenship behavior directed toward individuals (8 items; e.g., “Help others who have been absent”) and organizational citizenship behavior directed toward the organization (8 items; e.g., “Express loyalty toward the organization”). EFA using principal axis factoring (PAF) with promax rotation supported a two-factor structure (KMO = 0.90; Bartlett’s χ^2^(78) = 3393.89, *p* < 0.001), explaining 53.98% of the variance. Factor loadings were strong for organizational citizenship behavior directed toward individuals (0.62–0.81) and the organization (0.59–0.80). Items 9, 10, and 11 were removed due to low loadings (< 0.50). CFA with the remaining 13 items confirmed good model fit (χ^2^ = 179.85, df = 64, χ^2^/df = 2.81, *p* = 0.000, TLI = 0.95, CFI = 0.96, RMSEA = 0.05). Reliability was satisfactory (α = 0.87 overall; 0.89 for OCBI; 0.85 for OCBO), confirming strong internal consistency.

#### 3.3.4 Mediating and moderating variables

Given the potential influence of lecturers’ demographic characteristics on organizational citizenship behavior and work engagement, this study incorporated both mediating and moderating variables into the research model. Specifically, work engagement was specified as a mediating variable to explain the underlying psychological mechanism linking authentic leadership to organizational citizenship behavior. In addition, education level and teaching experience were examined as moderating variables to assess whether the strength of these relationships varies across different professional backgrounds.

### 3.5 Data collection procedure

The Mekong Delta provides a meaningful context for examining authentic leadership, work engagement, and organizational citizenship behavior due to its Confucian-influenced higher education system, characterized by high power distance, hierarchical governance, and centralized authority (Hallinger et al., 2017; Truong et al., 2017). As authentic leadership theory was developed primarily in Western contexts (Walumbwa et al., 2008), this setting offers a valuable test of its cross-cultural applicability (Zhang et al., 2022). Ongoing institutional reforms and increasing performance demands further highlight the need for leadership that fosters intrinsic motivation and discretionary behaviors. Data were collected from full-time lecturers at 10 public universities in the Mekong Delta. Participants were randomly invited following institutional approval and received information about the study’s purpose and voluntary nature. Informed consent was obtained, and anonymity and confidentiality were ensured. The survey included validated measures of authentic leadership, work engagement, organizational citizenship behavior, and demographic variables. Data were collected through online and paper-based formats, with an average completion time of approximately 20 min.

### 3.6 Data analysis

Data analysis was conducted in four stages using SPSS (version 25) and AMOS. First, data screening ensured suitability for multivariate analysis. Missing data were examined and addressed using listwise deletion when minimal, or the expectation–maximization (EM) method when necessary to preserve statistical power (Hair et al., 2014; Kline, 2016). Descriptive statistics (means and standard deviations) were computed to summarize key variables. Second, EFA using principal axis factoring with promax rotation was performed to identify underlying factor structures. Sampling adequacy was assessed via the Kaiser–Meyer–Olkin (KMO) measure and Bartlett’s test of sphericity, with thresholds of KMO > 0.50 and *p* < 0.05, respectively. Factors were retained based on eigenvalues > 1 and loadings ≥ 0.50, with poorly loading items removed (Hair et al., 2006; Tabachnick and Fidell, 2019). Third, CFA using maximum likelihood estimation in AMOS validated the measurement model. Model fit was evaluated using χ^2^/df ≤ 3 (or ≤ 5), CFI and TLI ≥ 0.90, and RMSEA ≤ 0.08 (Hu and Bentler, 1999; Steiger, 2007). Reliability was assessed using Cronbach’s alpha (≥ 0.70) (Nunnally and Bernstein, 1994). Finally, hypotheses were tested using Hayes’ PROCESS macro. Direct and mediating effects were examined with Model 4 (5,000 bootstrap samples; 95% confidence intervals), while moderating effects were tested using Model 1.

## 4 Results

### 4.1 Descriptive statistics

Prior to hypothesis testing, preliminary analyses were conducted to assess the psychometric properties of the measurement scales and to examine the bivariate relationships among the study variables. Internal consistency reliability was evaluated using Cronbach’s alpha (α). The results indicated acceptable to good reliability for all constructs, including authentic leadership (α = 0.82), work engagement (α = 0.82), and organizational citizenship behavior (α = 0.87). Descriptive statistics and Pearson product–moment correlation coefficients are presented in Table 2. The mean scores for all variables were above the midpoint of the scale, suggesting generally positive perceptions of authentic leadership, work engagement, and organizational citizenship behavior among respondents. Correlation analysis revealed that all three variables were significantly and positively related to one another. Specifically, authentic leadership was positively associated with work engagement (*r* = 0.64, *p* < 0.01) and organizational citizenship behavior (*r* = 0.52, *p* < 0.01), while work engagement was also positively correlated with organizational citizenship behavior (*r* = 0.51, *p* < 0.01). In addition, preliminary analyses indicated that age and gender were not significantly related to the focal study variables (all *p* > 0.05). Therefore, these demographic variables were not included as control variables in the subsequent PROCESS analyses.

### 4.2 Testing research hypotheses

**FIGURE 2:**
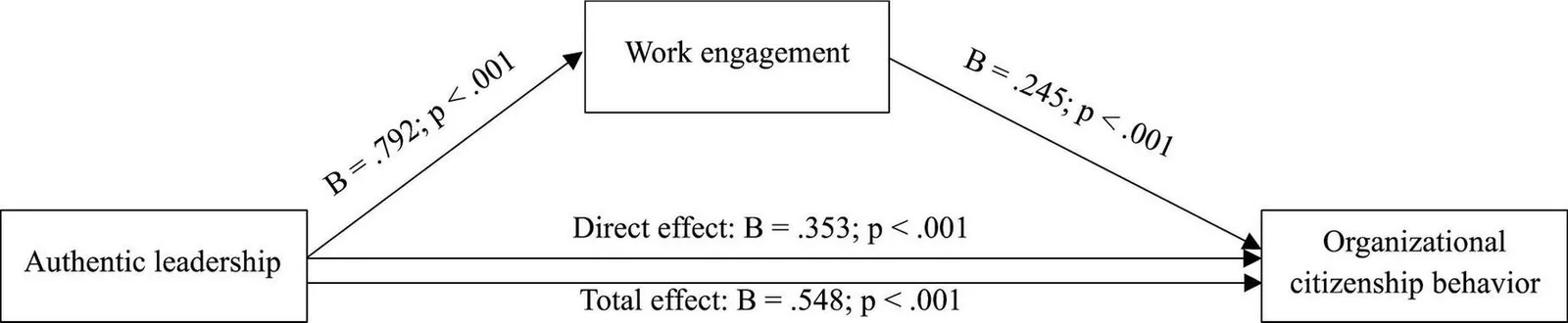
Results of the mediation analysis.

The results presented in Table 3 and Figure 2 indicate that two of the four hypotheses were supported. Authentic leadership had a significant positive effect on organizational citizenship behavior, supporting Hypothesis 1. Work engagement also significantly mediated this relationship, supporting Hypothesis 2. However, the moderating effects of education level and work experience were not significant. These findings indicate that authentic leadership influences organizational citizenship behavior both directly and indirectly through engagement, while its effectiveness remains consistent across different levels of education and work experience.

**TABLE 3:** Descriptive statistics, and correlations among study variables.

Variable	M	SD	AL	WE	OCB
AL	3.24	0.39	–	–	–
WE	3.19	0.47	0.645**		
OCB	3.15	0.40	0.526**	0.509**	

AL, Authentic leadership; OCB, organizational citizenship behavior; WE, work engagement.

**p < 0.01 (two-tailed).

#### 4.2.1 The main effect

Hypothesis 1 proposed that authentic leadership is positively associated with the organizational citizenship behavior of faculty members. The results presented in Table 4 show that authentic leadership had a significant and positive effect on organizational citizenship behavior (*B* = 0.548, *p* < 0.001). This indicates that higher levels of perceived authentic leadership are associated with greater engagement in citizenship behaviors. Specifically, when leaders demonstrate self-awareness, ethical integrity, relational transparency, and balanced decision-making, faculty members are more likely to exhibit discretionary behaviors that support colleagues and contribute to institutional effectiveness. The results provide strong empirical support for Hypothesis 1, confirming that authentic leadership is a significant predictor of organizational citizenship behavior in higher education contexts.

**TABLE 4:** Results of the mediation analysis.

Variable	B	SE	*t*	*p*	LLCI	ULCI	95% Boot CI
Direct effects (outcome: OCB)							
Constant	1.226	0.122	10.016	< 0.001	0.986	1.467	
AL	0.353	0.048	7.339	< 0.001	0.259	0.448	
WE	0.245	0.039	6.265	< 0.001	0.168	0.322	
Total effect							
AL → OCB	0.548	0.038	14.379	< 0.001	0.473	0.622	
Indirect effect							
AL → WE → OCB	0.194	0.034					[0.130, 0.264]

*R*^2^ = 0.326, Adjusted *R*^2^ = 0.323, *F*(2, 539) = 130.326, *p* < 0.001. *B*, unstandardized regression coefficient; SE, standard error; *t*, *t*-value; *p*, *p*-value; CI, confidence interval; LLCI, lower limit CI; ULCI, upper limit CI. Bootstrap confidence intervals based on 5,000 samples. Indirect effect CI does not include zero, indicating significant mediation. AL, Authentic leadership; OCB, organizational citizenship behavior; WE, work engagement.

#### 4.2.2 The mediated effect

Hypothesis 2 posited that work engagement mediates the positive association between authentic leadership and organizational citizenship behavior. The mediation analysis was conducted using Hayes’ PROCESS macro with bootstrap resampling (5,000 samples). As shown in Table 4, when work engagement was included in the model, authentic leadership remained a significant predictor of organizational citizenship behavior (*B* = 0.353, *p* < 0.001), while work engagement also significantly predicted organizational citizenship behavior (*B* = 0.245, *p* < 0.001). These results indicate that both direct and indirect pathways are significant. The indirect effect of authentic leadership on organizational citizenship behavior through work engagement was statistically significant (*B* = 0.194, 95% CI [0.130, 0.264]), as the confidence interval did not include zero. This finding confirms the presence of partial mediation, suggesting that authentic leadership influences organizational citizenship behavior both directly and indirectly by enhancing employees’ work engagement. Accordingly, Hypothesis 2 was supported.

#### 4.2.3 The moderated effects

Hypothesis 3 proposed that education level moderates the association between authentic leadership and work engagement, such that the association is stronger for employees with higher levels of education. The results of the moderation analysis are presented in Table 5. As shown, authentic leadership had a significant positive effect on work engagement (*B* = 0.874, *p* < 0.001). However, education level was not a significant predictor of work engagement (*B* = 0.121, *p* = 0.498). More importantly, the interaction term between authentic leadership and education level was not statistically significant (*B* = −0.038, *p* = 0.475, 95% CI [−0.141, 0.066]). The change in explained variance due to the interaction effect was negligible (Δ*R*^2^ = 0.001, *p* = 0.475), indicating that education level does not significantly alter the strength of the relationship between authentic leadership and work engagement. These findings suggest that the positive influence of authentic leadership on work engagement is consistent across different educational levels. Therefore, Hypothesis 3 was not supported.

**TABLE 5:** Results of the moderation analysis.

Predictor	*B*	SE	*t*	*p*	LLCI	ULCI
AL	0.874	0.122	7.190	< 0.001	0.635	1.113
Education level	0.121	0.178	0.677	0.498	−0.229	0.470
AL × Education level (Interaction)	−0.038	0.052	−0.716	0.475	−0.141	0.066
R2= 0.416, F(3, 538) = 127.762, p < 0.001; dependent variable = WE						
Predictor						
AL	0.441	0.108	4.091	< 0.001	0.229	0.652
Work experience	−0.137	0.142	−0.967	0.334	−0.416	0.141
AL × Work experience (Interaction)	0.047	0.043	1.074	0.283	−0.039	0.132

*R*^2^ = 0.279, *F*(3, 538) = 69.497, *p* < 0.001; Dependent variable = OCB. B, unstandardized regression coefficient; SE, standard error; *t*, *t*-value; *p*, *p*-value; CI, confidence interval; LLCI, lower limit CI; ULCI, upper limit CI. AL, Authentic leadership; OCB, organizational citizenship behavior; WE, work engagement.

Hypothesis 4 proposed that work experience moderates the association between authentic leadership and organizational citizenship behavior, such that the association is stronger for employees with higher levels of work experience. The results of this analysis are presented in Table 4. Authentic leadership was found to have a significant positive effect on organizational citizenship behavior (*B* = 0.441, *p* < 0.001). However, work experience was not a significant predictor of organizational citizenship behavior (*B* = −0.137, *p* = 0.334). Furthermore, the interaction between authentic leadership and work experience was not statistically significant (*B* = 0.047, *p* = 0.283, 95% CI [−0.039, 0.132]). The inclusion of the interaction term resulted in only a trivial increase in explained variance (Δ*R*^2^ = 0.002), indicating a lack of moderating effect. These findings indicate that the relationship between authentic leadership and organizational citizenship behavior does not vary significantly across levels of work experience. Accordingly, Hypothesis 4 was not supported.

## 5 Discussion

The findings provide support for the central argument that authentic leadership promotes organizational citizenship behavior among university lecturers, both directly and indirectly through work engagement. Overall, the results support the integration of theories. However, the proposed boundary conditions based on education level and work experience were not supported.

A significant positive relationship was found between authentic leadership and organizational citizenship behavior, suggesting that lecturers who perceived their leaders as transparent, ethical, balanced, and self-aware tended to report greater engagement in discretionary behaviors that benefit colleagues and the institution. This finding is consistent with authentic leadership theory, which argues that authentic leaders foster trust, moral consistency, and positive leader–follower relationships (Avolio and Gardner, 2005; Gardner et al., 2011; Walumbwa et al., 2008). It also aligns with social exchange theory, because lecturers may reciprocate fair and ethical leadership by contributing beyond formal role expectations (Blau, 1964; Cropanzano and Mitchell, 2005). Empirically, this result is consistent with prior studies demonstrating positive relationships between authentic leadership and organizational citizenship behavior across sectors and cultural contexts (Alvarez et al., 2019; Farid et al., 2020; Hirst et al., 2016; Jun et al., 2023; Shapira-Lishchinsky and Tsemach, 2014; Shie and Chang, 2022). Moreover, meta-analytic evidence confirms that authentic leadership is a robust predictor of positive employee behaviors, including citizenship behaviors (Zhang et al., 2022).

Work engagement significantly mediated the relationship between authentic leadership and organizational citizenship behavior. This suggests that authentic leadership not only exerts a direct influence on citizenship behavior but also enhances lecturers’ vigor, dedication, and absorption, which subsequently promote discretionary contributions. This finding is strongly aligned with the job demands–resources model, where leadership is conceptualized as a key job resource that fosters motivational states (Bakker and Demerouti, 2007, 2017; Bakker et al., 2011). It also corroborates previous studies demonstrating the mediating role of work engagement in linking authentic leadership to positive outcomes such as performance, creativity, and organizational citizenship behavior (Chaudhary and Panda, 2018; Lv et al., 2022; Wei et al., 2018). Additional research further supports the role of engagement as a psychological mechanism through which leadership influences employee attitudes and behaviors (Bakker, 2022; Hsieh and Wang, 2015; Miraflor and Lyndon, 2022). The partial mediation observed in this study suggests that engagement is a key, but not exclusive, mechanism. Other mediators such as trust, identification, and psychological empowerment have also been identified in prior research (Farid et al., 2020; Joo and Jo, 2017; Leroy et al., 2015).

Education level did not significantly moderate the relationship between authentic leadership and work engagement. While prior literature suggests that education can influence cognitive complexity, value orientation, and responsiveness to leadership (Ariffin and Norbani Che, 2014; Hadi et al., 2023; Hitka et al., 2021; Hoefsmit et al., 2023), the current findings indicate that the positive association between authentic leadership and work engagement is relatively consistent across educational levels. One plausible explanation is that the sample consists entirely of university lecturers who operate within a relatively homogeneous professional environment characterized by shared expectations regarding academic professionalism, ethical conduct, and collegiality. More importantly, the finding may reflect the influence of the Confucian cultural context emphasized in this study. Confucian traditions place strong emphasis on moral leadership, integrity, reciprocity, respect for authority, and harmonious social relationships (Hallinger et al., 2017; Nguyen et al., 2015; Truong et al., 2017). Because authentic leadership embodies many of these characteristics through self-awareness, relational transparency, balanced decision-making, and an internalized moral perspective (Avolio and Gardner, 2005; Gardner et al., 2011; Walumbwa et al., 2008), lecturers with different educational backgrounds may respond similarly to authentic leaders. Furthermore, emerging evidence from Vietnamese and other Confucian-influenced settings suggests that leadership behaviors reflecting ethical consistency and relational harmony are widely valued and positively associated with employee engagement and commitment (Nguyen et al., 2024; Yen et al., 2024). Therefore, authentic leadership may function as a culturally congruent leadership resource whose effectiveness transcends educational differences.

Work experience did not significantly moderate the relationship between authentic leadership and organizational citizenship behavior. Although prior research suggests that experience shapes employees’ cognitive schemas and interpretations of leadership behaviors (Hirst et al., 2016; Li et al., 2022; Liu et al., 2018), the findings indicate that authentic leadership is positively associated with organizational citizenship behavior across different career stages. This pattern may also be understood through the cultural characteristics of Vietnamese higher education institutions. Confucian values emphasize collective responsibility, social harmony, loyalty to the group, and the fulfillment of obligations toward the broader community (Hallinger et al., 2017; Nguyen et al., 2015; Truong et al., 2017). These values may encourage both less experienced and more experienced lecturers to respond positively to leaders who demonstrate ethical conduct, fairness, and concern for others. From a social exchange perspective, authentic leaders establish relationships characterized by trust and mutual respect, encouraging employees to reciprocate through citizenship behaviors regardless of their tenure or professional experience (Blau, 1964; Cropanzano and Mitchell, 2005). Moreover, ongoing reforms in Vietnamese higher education have increased expectations regarding accountability, collaboration, and institutional commitment across academic ranks and career stages (Le, 2024; Nguyen T. D. et al., 2023). Consequently, authentic leadership may provide a common relational and ethical foundation that promotes citizenship behavior among faculty members irrespective of their work experience.

These findings extend the broaden-and-build theory (Fredrickson, 2001) in a more nuanced manner. While the study hypothesized that education level and work experience would strengthen leadership effects by enhancing employees’ personal resources, the results suggest that authentic leadership itself may function as a broadly shared psychological and relational resource. By fostering positive emotions, trust, and high-quality interpersonal relationships (Agote et al., 2016; Braun and Peus, 2018), authentic leadership appears to encourage engagement and organizational citizenship behavior across demographic groups. More importantly, the findings contribute to the growing literature examining authentic leadership in non-Western contexts. Although authentic leadership was primarily developed and tested in Western settings (Polat et al., 2024), the present study suggests that its core principles are highly compatible with the values embedded in Confucian cultures. The non-significant moderating effects of education level and work experience imply that the benefits of authentic leadership are not restricted to particular demographic groups but may be broadly shared among university lecturers. In the Vietnamese context, where leadership relationships are shaped by respect for moral authority, interpersonal obligations, relational harmony, and collective wellbeing (Hallinger et al., 2017; Nguyen et al., 2015; Truong et al., 2017), authentic leadership may be perceived as a legitimate and desirable leadership approach across educational and experiential backgrounds. Thus, rather than weakening the cultural argument, the absence of demographic differences may itself reflect the strength of shared cultural values that shape how leadership is interpreted and reciprocated. This finding contributes to the cross-cultural validation of authentic leadership and supports its applicability within Confucian, high-power-distance higher education settings undergoing institutional transformation (Le, 2024; Nguyen T. D. et al., 2023; Nguyen et al., 2024). Accordingly, authentic leadership appears to represent a broadly effective mechanism for fostering work engagement and organizational citizenship behavior among university faculty members.

## 6 Practical implications

The findings offer several important implications for leadership practice and policy in higher education institutions, particularly within Confucian and high power-distance contexts such as Vietnam (Hallinger et al., 2017; Truong et al., 2017). In such environments, where hierarchical relationships and respect for authority are deeply embedded, leadership approaches that emphasize ethics, transparency, and relational integrity become especially critical for fostering positive employee attitudes and behaviors.

First, the results underscore the central role of authentic leadership in promoting organizational citizenship behavior. University administrators and policymakers should therefore prioritize the development of leadership competencies grounded in self-awareness, ethical decision-making, relational transparency, and balanced processing, which constitute the core dimensions of authentic leadership (Avolio and Gardner, 2005; Walumbwa et al., 2008). These competencies are not merely abstract ideals but can be systematically developed through structured leadership development programs, executive training, and mentoring initiatives. Embedding these principles into leadership pipelines is particularly important because authentic leadership has been empirically linked to increased trust, positive emotional experiences, and discretionary behaviors such as organizational citizenship behavior (Agote et al., 2016; Farid et al., 2020; Shie and Chang, 2022; Zhang et al., 2022). As universities face growing pressures for accountability and performance, fostering such leadership qualities can contribute to a more ethical, collaborative, and high-performing academic environment.

Second, the significant mediating role of work engagement highlights the importance of focusing not only on observable behaviors but also on the underlying psychological states of academic staff. Specifically, institutions should aim to enhance lecturers’ vigor, dedication, and absorption key components of work engagement (Schaufeli et al., 2002; Schaufeli and Bakker, 2010). Drawing on the job demands–resources framework, leadership can be viewed as a critical job resource that energizes employees and sustains motivation (Bakker and Demerouti, 2007, 2017; Bakker, 2022). Accordingly, universities should invest in creating supportive work environments that promote autonomy, recognize academic contributions, and provide adequate resources for teaching and research. Such initiatives are particularly relevant in higher education, where intrinsic motivation plays a vital role. Prior research demonstrates that higher levels of engagement are associated with improved teacher performance, wellbeing, self-efficacy, and overall organizational effectiveness (Burić and Macuka, 2018; Salanova et al., 2005; Tran Q. H. N, 2023; Wang, 2022). Thus, strengthening engagement represents a key pathway through which leadership can influence institutional outcomes.

Third, the absence of significant moderating effects of education level and work experience suggests that the positive associations among authentic leadership, work engagement, and organizational citizenship behavior are relatively consistent across diverse faculty groups. This finding has practical value, as it indicates that universities may not need highly differentiated leadership strategies based on demographic characteristics. Instead, institutions can focus on implementing a consistent, authenticity-based leadership approach across all levels of academic management. This is particularly relevant in the Vietnamese higher education system, where leadership practices are influenced by both traditional hierarchical norms and ongoing institutional reforms aimed at enhancing autonomy and global competitiveness (Le, 2024; Nguyen et al., 2015; Nguyen T. D. et al., 2023). A unified leadership approach grounded in authenticity may help bridge these dual pressures by fostering both stability and adaptability.

Finally, in the broader context of higher education reform in Vietnam, authentic leadership offers a sustainable and human-centered approach to institutional development. By strengthening trust, engagement, and voluntary contributions, it can enhance collaboration, knowledge sharing, and long-term organizational resilience. This is especially important as universities navigate complex challenges such as digital transformation, internationalization, and increasing performance expectations. Prior research in Vietnam has already highlighted the importance of leadership in shaping teacher outcomes and organizational effectiveness (Maheshwari, 2021; Trinh et al., 2025; Wirawan et al., 2020). Building on these findings, the present study suggests that authentic leadership can serve as a foundational mechanism for fostering not only performance but also a positive and sustainable academic culture.

## 7 Limitations and future research

Despite its contributions, this study has several limitations that suggest directions for future research. First, the cross-sectional design limits causal inference among authentic leadership, work engagement, and organizational citizenship behavior. Future studies should employ longitudinal or experimental designs to better examine temporal relationships and explore how leadership perceptions, engagement, and citizenship behaviors evolve over time. Second, reliance on self-reported data raises concerns regarding common method bias and social desirability. Although Harman’s single-factor test and the combined CFA suggested that common method bias was unlikely to pose a serious threat, future research could adopt multi-source approaches, such as incorporating supervisor or peer evaluations of organizational citizenship behavior, as well as objective performance indicators. Multi-level designs examining leadership effects at individual, team, and organizational levels would further enhance methodological rigor. Third, although the combined CFA demonstrated an acceptable fit for the hypothesized three-factor measurement model, this study did not compare the proposed model with alternative measurement models (e.g., two-factor or one-factor models). Therefore, evidence regarding the distinctiveness of the study constructs should be interpreted with some caution. Future research should conduct more comprehensive assessments of discriminant validity through alternative model comparisons and additional techniques, such as the heterotrait–monotrait (HTMT) ratio or related measurement validation procedures. Fourth, the sample was restricted to public universities in the Mekong Delta region of Vietnam, limiting the generalizability of the findings. Future studies should replicate the research across diverse institutional contexts, including private universities and institutions located in other regions, and conduct cross-cultural comparisons to examine the applicability of authentic leadership in different cultural environments. Fifth, this study examined only education level and work experience as moderators. Future research should explore additional boundary conditions, such as psychological capital, career stage, organizational culture, leadership climate, and person–organization fit. Given the non-significant moderating effects observed in this study, future research may also investigate whether cultural values, professional identity, or organizational characteristics serve as more salient boundary conditions in explaining leadership effectiveness. Finally, although work engagement was identified as an important mediating mechanism, other explanatory processes, including trust, leader–member exchange, psychological empowerment, organizational identification, and public service motivation, warrant further investigation. Expanding outcome variables to include innovation, employee wellbeing, job performance, and turnover intention would provide a more comprehensive understanding of the mechanisms and consequences of authentic leadership.

## 8 Conclusion

This study examined the relationship between authentic leadership and organizational citizenship behavior among university lecturers in Southern Vietnam, with work engagement as a mediating mechanism and education level and work experience as moderating variables. The findings indicate that authentic leadership is significantly and positively associated with organizational citizenship behavior, suggesting that lecturers who perceive their leaders as demonstrating ethical integrity, transparency, self-awareness, and balanced decision-making also report higher levels of voluntary and constructive behaviors. The results further support the mediating role of work engagement in the relationship between authentic leadership and organizational citizenship behavior, indicating that authentic leadership is positively associated with lecturers’ vigor, dedication, and absorption, which are in turn associated with higher levels of citizenship behavior. However, the moderating effects of education level and work experience were not supported, suggesting that the relationships examined in this study are relatively consistent across demographic groups. These findings highlight the relevance of authentic leadership for fostering work engagement and organizational citizenship behavior in higher education, particularly within a Confucian, high power-distance context. The study contributes to the educational leadership and organizational behavior literature by extending understanding of authentic leadership in a non-Western setting and identifying work engagement as an important explanatory mechanism underlying this relationship. Practically, the findings offer insights for promoting trust, collaboration, and institutional effectiveness in universities undergoing reform and facing increasing performance demands.

## Data Availability

The original contributions presented in the study are included in the article/supplementary material, further inquiries can be directed to the corresponding author.
